# Detailed Analysis of the Microbial Population in Malaysian Spontaneous Cocoa Pulp Fermentations Reveals a Core and Variable Microbiota

**DOI:** 10.1371/journal.pone.0081559

**Published:** 2013-12-16

**Authors:** Esther Meersman, Jan Steensels, Melissa Mathawan, Pieter-Jan Wittocx, Veerle Saels, Nore Struyf, Herwig Bernaert, Gino Vrancken, Kevin J. Verstrepen

**Affiliations:** 1 Laboratory for Genetics and Genomics, Centre of Microbial and Plant Genetics (CMPG), KU Leuven, Leuven, Belgium; 2 Laboratory for Systems Biology, Vlaams Instituut voor Biotechnologie (VIB), Leuven, Belgium; 3 Barry Callebaut AG, Zurich, Switzerland; Louisiana State University, United States of America

## Abstract

The fermentation of cocoa pulp is one of the few remaining large-scale spontaneous microbial processes in today's food industry. The microbiota involved in cocoa pulp fermentations is complex and variable, which leads to inconsistent production efficiency and cocoa quality. Despite intensive research in the field, a detailed and comprehensive analysis of the microbiota is still lacking, especially for the expanding Asian production region. Here, we report a large-scale, comprehensive analysis of four spontaneous Malaysian cocoa pulp fermentations across two time points in the harvest season and two fermentation methods. Our results show that the cocoa microbiota consists of a “core” and a “variable” part. The bacterial populations show a remarkable consistency, with only two dominant species, *Lactobacillus fermentum* and *Acetobacter pasteurianus*. The fungal diversity is much larger, with four dominant species occurring in all fermentations (“core” yeasts), and a large number of yeasts that only occur in lower numbers and specific fermentations (“variable” yeasts). Despite this diversity, a clear pattern emerges, with early dominance of apiculate yeasts and late dominance of *Saccharomyces cerevisiae*. Our results provide new insights into the microbial diversity in Malaysian cocoa pulp fermentations and pave the way for the selection of starter cultures to increase efficiency and consistency.

## Introduction

The curing of cocoa beans under influence of the cocoa pulp fermentation, the first step in the production of chocolate, is one of the few remaining large-scale spontaneous microbial processes in the food industry. Previous reports indicate that yeasts, lactic acid bacteria (LAB) and acetic acid bacteria (AAB) are the key players of this fermentation process, during which the mucilaginous, sugary pulp that surrounds the beans is liquefied [1], [2]. Together, these microorganisms are responsible for the generation of ethanol, lactic acid, acetic acid and heat during the fermentation and for an increased air penetration as the fermentation proceeds [3], [4]. However, despite this general description of cocoa pulp fermentations, there is considerable variation between different fermentations, because the spontaneous nature of cocoa pulp fermentations leads to variations in the active microbiota. This in turn has been suggested to give rise to undesirable variability in product quality [1].

Because of the variability associated with spontaneous fermentations, almost all bulk food fermentation processes, including the production of beer, wine and bread, are nowadays inoculated with a defined starter culture to obtain reproducible fermentation parameters and consistent product quality. However, since spontaneously fermented foodstuffs, such as specialized beers, wine and other beverages, still represent a significant part of the production market, the microbiota involved in these fermentations remains a topic of interest [5], [6], [7], [8], [9].

Several studies have investigated the microbial diversity of spontaneous cocoa pulp fermentations [10], [11], [12], [13], [14], [15], [16], [17], [18]. Most of these research papers rely on culture-dependent techniques, i.e. plating and subsequent identification of single colonies. Since there are several potential drawbacks of this approach (such as media bias and no detection of uncultivable species), alternative molecular methods [such as denaturing gradient gel electrophoresis (DGGE)] have been described [19], [20]. Because labor-intensive identification of single isolates is avoided, these methods can serve as a fast source of information of the global population structure, and can detect the presence of (even uncultivable) species. However, comparative analyses between both culture-dependent and -independent approaches in spontaneous cocoa pulp fermentation indicate that results obtained with both techniques are comparable, with (generally) a higher biodiversity detected with culture-dependent approaches [13], [17]. Additionally, when aiming to obtain micro-organisms to apply in a defined starter culture, culture-dependent approaches are the only strategy that will yield a broad collection of indigenous micro-organisms, that afterwards can be characterized and used as inocula. Therefore, the use of a large-scale culture-dependent approach combined with accurate identification methods such as ribosomal DNA (rDNA) sequencing or DNA fingerprinting [e.g. repetitive DNA sequence-based PCR (rep-PCR), or amplification fragment length polymorphism (AFLP)] will yield both a detailed mapping of the (diversity in the) microbial population and allow isolation of pure cultures that can (after characterization) potentially be applied as starter cultures [21].

More recently, novel interesting microbial population profiling methods using direct sequencing of mixed communities (metagenomics) are developed that allow high-throughput and in-depth analysis of the microbiota of spontaneous fermentation processes [8], [22]. This approach has proven to be extremely valuable to identify microbial subgroups which are not specifically targeted by most culture-dependent approaches (e.g. *γ-Proteobacteria* [22]). Potential disadvantages of this approach include the dependency on the extraction method (which can influence the detected population structure [23]) and low discriminative power between species due to the lack of appropriate primers, although much recent research focuses on automated primer design and/or optimization [24], [25]. Nevertheless, this upcoming strategy holds great potential to analyze the full microbial population structure in many different environments, such as spontaneous fermentations [8], [22].

Although spontaneous cocoa pulp fermentations have been investigated in different geographical locations and using different experimental setups, the main microbial subgroups driving the fermentation process are always identified as yeasts, LAB and AAB. Yeasts produce pectinolytic enzymes that break down the cocoa pulp and convert the available sugars into ethanol, organic acids and volatile (aroma) compounds [1], [3]. Yeast species from the genera *Candida*, *Cryptococcus*, *Debaryomyces*, *Eremothecium*, *Hanseniaspora*, *Kluyveromyces*, *Kodamaea*, *Lodderomyces*, *Meyerozyma*, *Pichia*, *Rhodotorula*, *Saccharomyces*, *Saccharomycopsis*, *Schizosaccharomyces*, *Torulaspora*, *Trichosporon*, *Vanderwaltozyma*, *Wickerhamomyces*, *Yarrowia* and *Zygosaccharomyces* have been detected in different cocoa pulp fermentations [2], [22], [26]. However, there is little consistency in the reported yeast genera and species [1], even for fermentations within the same country [12], [14].

Together with yeasts, LAB also occur primarily in the first phase of the fermentation. These microorganisms metabolize sugars and citric acid to produce lactic acid, acetic acid and ethanol [27]. Several species have been reported, most notably *Lactobacillus fermentum*, *Lactobacillus plantarum* and *Leuconostoc pseudomesenteroides* [10], [13], [14], [15], [16], [17], [18]. In some cases, other LAB, such as *Lactobacillus brevis*, *Leuconostoc mesenteroides*, *Enterococcus* spp. and *Weisella* spp. are also detected, but mostly at very low concentrations [13], [17].

AAB mainly occur in the second phase of the fermentation. They are responsible for the aerobic and exothermic oxidation of ethanol into acetic acid, resulting in a temperature increase that contributes to the inactivation of the plant embryo [1], [3]. The overall diversity in the AAB population in spontaneous cocoa pulp fermentations seems low, with *Acetobacter pasteurianus* being reported in all studies [10], [13], [14], [15], [16], [17], [18].

Despite intensive research in the field, some seminal questions remain unanswered. Most studies only focus on one time point in the harvest season and/or one type of microorganisms (bacteria or yeasts), and in many cases only one or a few samples are taken throughout the fermentation process. This makes it difficult to obtain a comprehensive picture of the cocoa pulp fermentation process and compare data of various studies to investigate if and how different fermentations differ from each other. Moreover, whereas it has been suggested that the microbiota might be influenced by the fermentation method (i.e. heap fermentations, where the cocoa beans are piled onto a heap on the ground; or box fermentations, where the beans are fermented in large boxes), only one study has directly investigated the effect of these two common fermentation systems on the bacterial diversity and consistency [18], whereas data for yeasts are still lacking. And lastly but most importantly, while most studies focus on the traditional cocoa growing regions of Western Africa or Southern America, detailed information about the fastly expanding Asian cocoa producing region is still limited [10], [28], [29], [30].

Here, we report a comprehensive study of the microbial diversity in multiple spontaneous, production-scale heap and box cocoa pulp fermentations during the main harvest season (2011–2012) in Malaysia, one of the most important producers in Asia. This setup allowed us to directly compare the microbiota between different harvest seasons and fermentation methods. This study is the first large-scale in-depth analysis in Asia that simultaneously analyses yeast and bacterial population structures, using highly accurate DNA sequencing methods for identification.

## Materials and Methods

### Cocoa pulp fermentations

A total of four cocoa pulp fermentations were set up at the Barry Callebaut cocoa research facility, Pahang, Malaysia during the main harvest season of 2011–2012. Two fermentations were performed in the beginning of the harvest season [box fermentation 1 (B1) and heap fermentation 1 (H1), October 2011], while the other two fermentations were executed at the end of the harvest season [box fermentation 2 (B2) and heap fermentation 2 (H2), January 2012]. Cocoa pods were harvested and opened after three days. Only healthy and mature pods were used for the fermentations and the placenta was removed after opening the pods. All equipment was thoroughly cleaned before every use. The plastic boxes used for the box fermentations had a volume of 800 kg of wet, freshly harvested cocoa beans and were washed with water prior to filling them. Fermentation volumes of 544 kg and 640 kg were respectively used for B1 and B2. The heaps were formed by placing the wet beans (292 kg for H1, 590 kg for H2) on banana leaves and covering the pile with additional banana leaves. The fermentations were treated according to local agricultural practices: B1 and H1 (executed during the early harvest season) were turned once after 48 h and stopped after 6 days by spreading the beans on a drying platform, while B2 and H2 (executed during the late harvest season) were turned twice, after 48 h and 96 h, and stopped after 5 days.

During three of the four fermentations, the temperature and pH were continuously measured in the middle of the fermenting mass using a digital pH meter (pH 3310 SET 2, SenTix® 41, WTW GmbH, Weilheim, Germany) (H1, B2 and H2) or measured manually for B1 at every sampling point at three standardized places in the middle of the box (pH 3310 SET 2, SenTix® 41, WTW GmbH, Weilheim, Germany).

### Sampling and analysis of the samples

Cocoa bean samples of the fermentations were taken after 0 (fresh cocoa beans, right after opening the pods), 6, 14, 24, 32, 48, 56, 72, 80, 96, 120 and 144 hours (last time point only for B1 and H1). All the samples were taken at the same depth of the fermenting bean mass (20 cm below the surface). Approximately 200 g of beans were aseptically taken, cooled to 4°C and further analyzed within 1 h.

Next, 40 g of the wet bean sample was mixed with 160 mL of 0.1% peptone water in an aseptic Stomacher bag. The suspension was powerfully shaken for 5 min in order to obtain a homogeneous cocoa pulp solution. A 10-fold serial dilution was made in 0.1% peptone water and 100 µl from each dilution was spread over three different selective agar plates. De Man-Rogosa-Sharpe medium (MRS) with 2% agar (incubated at 30°C for 2 days), supplemented with 0.01% cycloheximide was used to analyze LAB growth. Acetic acid medium (AAM) agar (42°C, 4 days), containing 1.5% peptone, 0.8% yeast extract, 1% glucose, 0.3% acetic acid, 0.5% ethanol, 3% agar and 0.01% cycloheximide was used to count and isolate AAB. Glucose was added in order to detect *Gluconobacter* spp., which cannot grow in culture media containing ethanol as a sole energy source [31]. Yeast counts were analyzed using yeast extract, peptone and dextrose medium (YPD) agar (30°C, 2 days), containing 1% yeast extract, 2% peptone, 2% glucose and 2% agar, supplemented with 0.01% chloramphenicol and 0.015% biphenyl. No yeast cell count could be obtained from the 24 h sample for H1, however, the yeast population dynamics and diversity was analyzed.

### Microbial isolation

Different strategies of picking bacterial and yeast isolates were used. For bacteria, three random isolates from each morphology present were picked from the countable plates for the different time points. The isolates obtained of the MRS and AAM agar plates were respectively grown in test tubes containing 3 mL MRS and AAM and stored at −20°C after adding 25% v v^−1^ glycerol. Per time point, 30 yeast isolates were randomly picked from the countable plates to analyze the population dynamics. Moreover, all morphologically different yeast colonies (including colonies on uncountable plates) were picked to investigate the full species diversity. Whenever the yeast count dropped below the detection limit [1.5 10^4^ colony forming units (CFU) g^−1^], all colonies were picked. The yeast isolates were grown in test tubes with 3 mL YPD and stored at −20°C after adding 25% v v^−1^ glycerol. A total number of 385 putative LAB isolates, 310 putative AAB isolates and 1033 putative yeast isolates were picked. All cryovials were transported to Belgium on dry ice to prevent thawing.

### Identification of bacterial isolates

In Belgium, all bacterial isolates were plated and checked for purity through successive transfer on AAM or MRS agar. Approximately 55% of the AAB isolates and 70% of the LAB isolates could be revived in Belgium. All bacterial isolates were identified using a polyphasic approach, starting with a phenotypic characterization (catalase and oxidase activity and Gram staining). Subsequently, bacterial DNA was extracted using InstaGene™ Matrix (Bio-Rad, Hercules, CA) to perform rep-PCR using the (GTG)_5_-primer [32], [33]. Cluster analysis of the fingerprints was achieved using BioNumerics software version 6.5 (Applied Maths, Sint-Martens-Latem, Belgium). The similarity between the rep-PCR fingerprints was expressed as percentage values of the Dice correlation coefficient and used for clustering by means of the unweighted pair group method with arithmetic mean (UPGMA) algorithm.

Only the rep-PCR fingerprints of the isolates that were suspected to be AAB or LAB according to the catalase and oxidase activity and Gram staining, were included in the dendrogram. Then, 16S rDNA gene sequences of 30 AAB and 88 LAB representatives of the different clusters were amplified using PCR. To identify the AAB isolates, the universal 16S rDNA primers 27F and 1492R were used [34], [35]. Amplification was carried out under the following conditions: initial denaturation at 95°C for 3 min; 35 cycles of denaturation at 95°C for 30 s, annealing at 50°C for 1 min and extension at 72°C for 1 min; and a final extension at 72°C for 7 min. For the LAB, LAC1 and LAC2 were used [36]. The following PCR-program was used: initial denaturation at 95°C for 5 min; 30 cycles of denaturation at 95°C for 20 s, annealing at 61°C for 45 s and extension at 72°C for 1 min; and a final extension at 72°C for 7 min. The PCR-product was purified and sequenced by VIB Genetic Service Facility (Antwerp, Belgium) using Applied Biosystems 3730XL DNA Analyzer. Sequencing data have been deposited in GenBank (KF738141–KF738146).

### Identification of yeast isolates

All 1033 putative yeast isolates were plated out and checked for purity through successive transfer on YPD agar in Belgium. Approximately 74% of the yeast isolates could be revived in Belgium.

Genomic DNA was extracted using zymolyase (Seikagaku Biobussiness, Tokyo, Japan). A single colony was dissolved into 50 µl of lysis solution [3 mg zymolyase (mL ultrapure water (Millipore, Billerica, MA))^−1^]. The solution was heated to 37°C for 60 min, followed by 10 min at 98°C. The variable D1/D2 domain of the large-subunit (26S) rDNA gene was amplified using primers NL-1 and NL-4, as described previously [37]. The PCR-product was purified and sequenced by VIB Genetic Service Facility (Antwerp, Belgium) using Applied Biosystems 3730XL DNA Analyzer.

The 26S rDNA gene sequence could not be used correctly identify *Candida metapsilosis*, *Candida orthopsilosis*, *Candida parapsilosis*, *Hanseniaspora opuntiae*, *Hanseniaspora thailandica* and *Pichia manshurica*, because of low or no sequence variability between closely related species. Therefore, the actin protein-coding (*ACT1*) region and the internally transcribed spacer (ITS) region of the rDNA gene cluster (ITS1-5.8S rDNA-ITS2) were additionally amplified for these isolates [38], [39], [40], [41], [42], [43]. The *ACT1* region was amplified using the primer pair CA21 and CA22R [39]. The primers ITS1-F [44] and ITS4 [45] were used to amplify the ITS region. The PCR-reactions were performed as described previously [39], [42]. Sequencing data have been deposited in GenBank (KF738147–KF738169).

## Results

To investigate the physical changes and microbial diversity in spontaneous Malaysian fermentations, we monitored four different production-scale fermentation processes, all differing in fermentation method and/or time point in the harvest season. By intensive sample taking, online monitoring of the fermentation parameters and microbial identification by rDNA sequencing, high-resolution data of the fermentation course could be deducted.

### Physical changes

All four fermentations started at similar temperatures (around 25°C), which first increased and then declined again over the course of the fermentations (Table 1). The temperature of B2 and H2, which were carried out later in the harvest season than B1 and H1, rose more quickly compared to B1 and H1, both before and after the first turn. Moreover, B2 and H2 were also turned a second time, resulting in a further increase of the temperature in H2 from 50.0°C to 52.6°C, while the temperature in B2 did not reach a new maximum value after the second turn.

**Table 1:** Temperature and pH of cocoa pulp in four Malaysian spontaneous cocoa pulp fermentations.

	pH				Temperature (°C)			
Fermentation*	pH_i_	pH_f_	pH_min_	pH_max_	T_i_	T_f_	T_min_	T_max_
B1	3.5	4.4	3.5	4.4	26.2	43.8	26.2	50.8
H1	3.5	4.4	3.4	4.4	26.2	45.5	26.2	47.0
B2	3.3	4.4	3.3	4.4	24.6	50.4	24.6	52.1
B2	3.8	4.4	3.4	4.5	25.8	49.2	25.8	52.6

*B1 and H1 (box and heap fermentation 1 respectively) were performed in the beginning of the harvest season (October 2011), while B2 and H2 (box and heap fermentation 2 respectively) were executed at the end of the harvest season (January 2012).

Different parameters are listed for each fermentation: pH_i_ = initial pH, pH_f_ = final pH, pH_min_ = minimal pH, pH_max_ = maximal pH, T_i_ = initial temperature, T_f_ = final temperature, T_min_ = minimal T, T_max_ =  maximal temperature.

The pulp surrounding the beans had an initial pH ranging from 3.3 (B2) to 3.8 (H2) (Table 1). The different pH profiles throughout the fermentations were comparable, except for the first 24 h, possibly because of differences in the fermentation setup, with the heap fermentations showing a more pronounced pH drop compared to the box fermentations.

### Microbiota analysis

**Figure 1:**
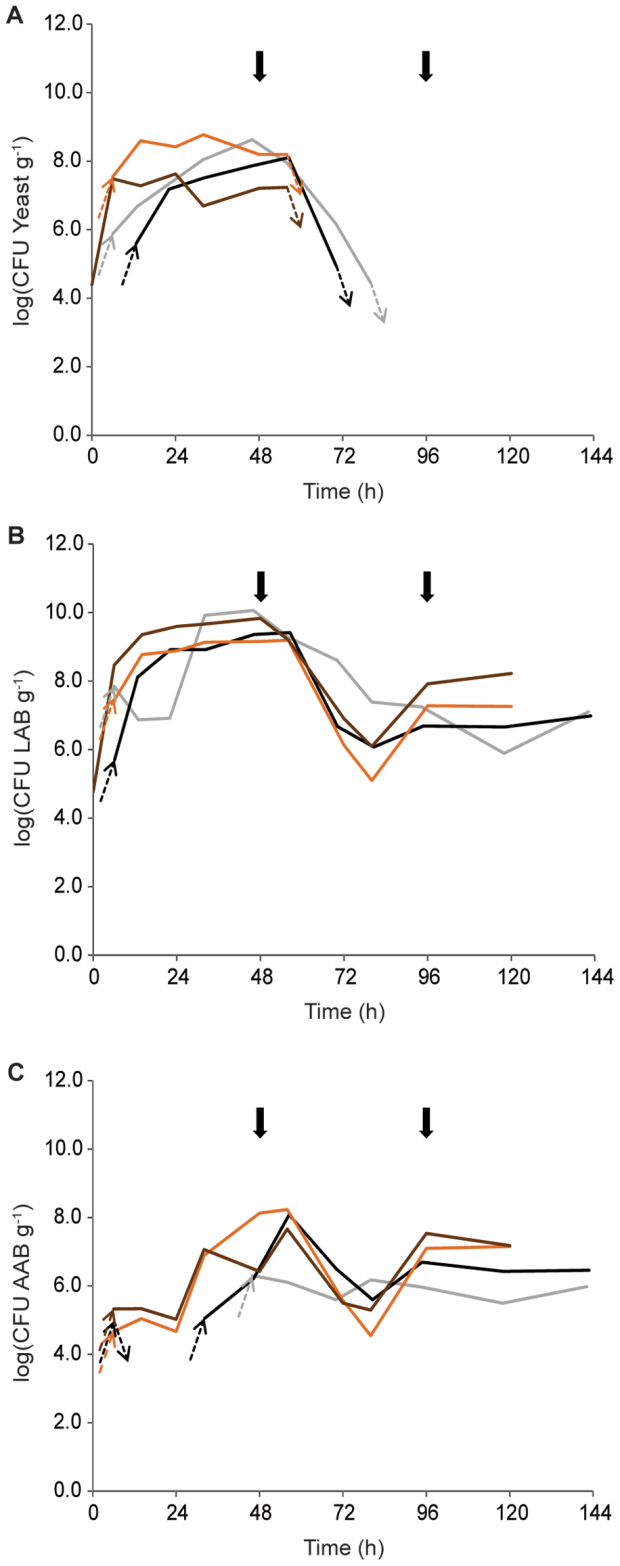
Microbial cell counts during four Malaysian spontaneous cocoa pulp fermentations. A: yeast. B: lactic acid bacteria (LAB). C: acetic acid bacteria (AAB). Box fermentation 1 (October 2011, black), heap fermentation 1 (October 2011, grey), box fermentation 2 (January 2012, brown) and heap fermentation 2 (January 2012, orange). The dashed arrows indicate when the cell counts rise up to or drop below the detection limit [1.5 10^4^ colony forming units (CFU) g^−1^]. Solid arrows indicate turning.

To quantify the microbial population of the four spontaneous Malaysian cocoa pulp fermentations, we first determined the total microbial counts of yeasts, LAB and AAB (Figure 1).

During three of the four fermentations, no yeasts were detected at the start of the fermentations. Only in B2, a low yeast concentration was detected in the first sample. In all fermentations, the yeast population showed a rapid growth phase during the first 24 h. After this initial surge, the yeast population remained high until 56 h, ranging from 4.9 10^6^ CFU g^−1^ to 5.8 10^8^ CFU g^−1^, followed by a rapid decline. The yeast population in the fermentations carried out at the end of the harvest season (B2, H2) disappeared faster compared to the fermentations performed at the start of the harvest season (B1, H1). In B1 and H1, yeasts were detected up until 80 h, while in B2 and H2, no yeasts were detected after 56 h, possibly because of the higher temperatures recorded in B2 and H2 (see above).

Similar to the yeast measurements, B2 was the only fermentation in which LAB were detected in the first sample. Similar trends in the LAB counts of all fermentations were observed, albeit within different time frames. There was a rapid increase in the LAB population during the first 14 h, reaching values of 1.3 10^8^ CFU g^−1^, 2.2 10^9^ CFU g^−1^ and 5.9 10^8^ CFU g^−1^ in B1, B2 and H2 respectively. In H1, the LAB population only started to increase notably after 22 h. After the initial rapid growth phase, the population remained relatively stable in all four fermentations, reaching maximal densities after 46 h to 56 h of 2.6 10^9^ CFU g^−1^, 1.2 10^10^ CFU g^−1^, 6.8 10^9^ CFU g^−1^ and 1.6 10^9^ CFU g^−1^ in B1, H1, B2 and H2, respectively. After 56 h, the LAB population started to decrease in all fermentations, which coincided with increasing temperatures after the first turn. When temperatures declined again, the LAB population densities increased again and then remained stable around 10^7^ CFU g^−1^ in all fermentations until the end. The LAB population reached higher cell counts compared to the yeast and AAB population at all time points throughout all fermentations.

In all fermentations, AAB densities only increased towards the end of the fermentation process. In B1, AAB were detected after 6 h, but subsequently dropped under the detection limit and were not detected until 32 h. In three of the four fermentations, the population reached its maximum size after 56 h, reaching comparable densities of 1.2 10^8^ CFU g^−1^, 4.6 10^7^ CFU g^−1^ and 1.7 10^8^ CFU g^−1^ in B1, B2 and H2, respectively. This increase is likely linked to the increase in aeration in the fermenting mass following the turning process, thereby facilitating the growth of aerobic AAB. Similar to the LAB population, the population density of AAB decreased between 56 h and 80 h in B1, B2 and H2. Subsequently, the population size increased to values around 10^7^ CFU g^−1^ and remained relatively stable until the end of the three fermentations. H1 showed a slightly aberrant AAB growth profile, with no AAB detected until 46 h.

### Bacterial diversity

Phenotypic analysis revealed that 196 out of 270 viable isolates (73%) from MRS agar were catalase negative, oxidase negative and Gram-positive (three characteristic properties of LAB), compared to 157 out of 170 viable isolates (92%) from AAM agar that were catalase positive, oxidase negative and Gram-negative (three characteristic properties of AAB).

To gain more detailed insight in the microbial diversity, we performed rep-PCR fingerprinting (see material and methods) on all putative LAB and AAB isolates. Analysis of the (GTG)_5_-PCR fingerprints of the 196 MRS isolates, combined with sequence analysis of the 16S rDNA of 88 representative isolates of the different clusters, confirmed that they were all correctly classified as LAB. A limited number of different LAB species was present, including *L. brevis*, *L. fermentum*, *L. plantarum*, *Lc. mesenteroides* and *Lc. pseudomesenteroides* (Table 2, Figure S1). *L. fermentum* and *L. plantarum* were the only two species isolated from all four fermentations. *L. fermentum* was the most prevalent LAB species in all fermentations and was present throughout the complete fermentation process. By contrast, *L. plantarum* was most often isolated in B1 and H1 but only played a minor role in B2 and H2. *Lc. mesenteroides* was not detected in B1, in H1 it was only detected in the first 14 h. This is in contrast to B2 and H2, carried out at the end of the harvest season, where *Lc. mesenteroides* represented the second biggest group of LAB after *L. fermentum*. *Lc. pseudomesenteroides* and *L. brevis* both were isolated sporadically and often at the start of the fermentations.

**Table 2:** Overview of all detected lactic acid and acetic acid bacterial species in the different spontaneous fermentations.

			Time point (h)										
Bacterial species	Fermentation*	Number of isolates	6	14	24	32	46	56	72	80	96	120	142
LAB													
*Lactobacillus brevis (1)*	B2	(1)						x					
*Lactobacillus fermentum (127)*	B1	(35)			x	x	x		x	x	x	x	x
	H1	(47)	x		x	x	x	x	x	x	x	x	x
	B2	(25)	x	x	x	x	x	x	x			x	
	H2	(20)		x	x	x	x	x	x	x	x	x	
*Lactobacillus plantarum (51)*	B1	(30)	x	x		x	x	x	x	x			x
	H1	(18)		x		x	x	x	x	x	x		
	B2	(1)		x									
	H2	(2)				x				x			
*Leuconostoc mesenteroides (13)*	H1	(4)	x										
	B2	(6)							x	x		x	
	H2	(3)								x	x	x	
*Leuconostoc pseudomesenteroides (4)*	H1	(2)	x			x							
	B2	(1)			x								
	H2	(1)				x							
AAB													
*Acetobacter pasteurianus (157)*	B1	(46)	x			x	x	x	x	x	x	x	x
	H1	(33)				x	x	x	x	x	x		x
	B2	(35)		x	x	x	x	x	x	x	x		
	H2	(43)	x	x	x	x	x	x	x	x	x	x	

*B1 and H1 (box and heap fermentation 1 respectively) were performed in the beginning of the harvest season (October 2011), while B2 and H2 (box and heap fermentation 2 respectively) were executed at the end of the harvest season (January 2012).

LAB = lactic acid bacteria, AAB = acetic acid bacteria.

The identification of 30 representative isolates of 157 putative AAB isolates confirmed that all putative isolates were all correctly classified as AAB, and that *A. pasteurianus* was the only AAB isolated from the fermentations (Table 2, Figure S2).

Together, these data indicate that in these Malaysian cocoa pulp fermentations, a conserved “core” bacterial microbiota exists, consisting of *L. fermentum* and *A. pasteurianus*.

### Diversity and population dynamics of the yeast population

**Figure 2:**
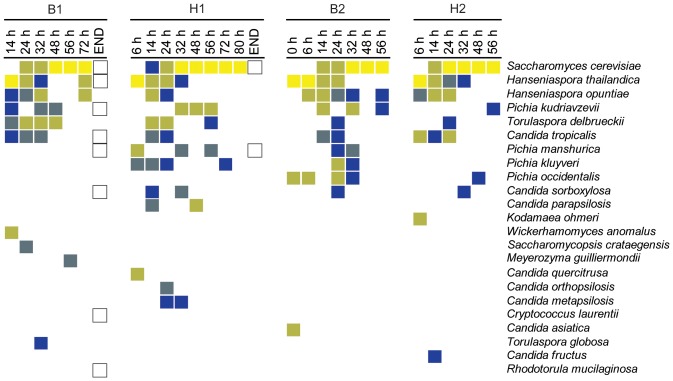
Yeast diversity in four Malaysian spontaneous cocoa pulp fermentations. Box fermentation 1 (B1, October 2011), heap fermentation 1 (H1, October 2011), box fermentation 2 (B2, January 2012) and heap fermentation 2 (H2, January 2012). In total, 769 yeast isolates were identified, allowing an in-depth analysis of the yeast diversity. Yeast species are ordered according to the frequency by which they were isolated from the different fermentation samples. The upper eight species were found in at least three fermentations, while the other species were only found in two or less fermentations. When the yeast counts were above the detection limit, different colors represent the relative population size of a certain species at a certain time point (dark blue : <5%, light blue : 5–10%, dark yellow : 10–50%, yellow : 50–100%). Hollow squares indicate that the yeast species was detected when the yeast cell count had dropped below the detection limit.

All four fermentations showed a large yeast diversity, with a total of 23 different yeast species detected (Figure 2). The largest diversity was found in B1, where 14 different yeast species were detected, compared to 13 species in H1, 11 in B2 and 10 in H2. *Candida sorboxylosa*, *Candida tropicalis*, *H. thailandica*, *H. opuntiae*, *Pichia kudriavzevii*, *Saccharomyces cerevisiae* and *Torulaspora delbrueckii* were generally present at higher densities and were detected in all four fermentations. Besides *P. kudriavzevii*, several other species from the genus *Pichia* were isolated, but all in lower concentrations. *P. manshurica* was only found in three fermentations (absent in H2). *Pichia occidentalis* and *Pichia kluyveri* were only detected in two fermentations, the former only being isolated from fermentations from the end of the harvest season. Further, a large number of yeast species was present at lower cell counts and only in one or a few fermentations, including *Kodamaea ohmeri*, *Wickerhamomyces anomalus*, *Saccharomycopsis crataegensis*, *Torulaspora globosa*, *Meyerozyma guilliermondii*, *Cryptococcus laurentii*, *Rhodotorula mucilaginosa* and several *Candida* species (Figure 2).

**Figure 3:**
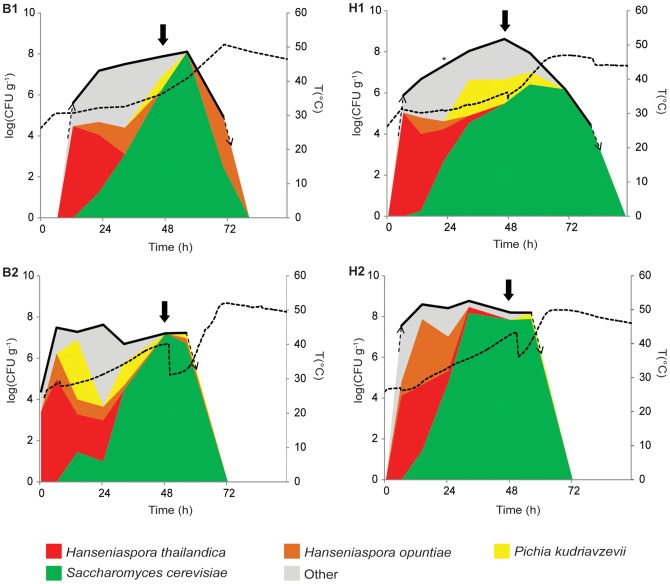
Yeast population dynamics in four Malaysian spontaneous cocoa pulp fermentations. Box fermentation 1 (B1, October 2011), heap fermentation 1 (H1, October 2011), box fermentation 2 (B2, January 2012) and heap fermentation 2 (H2, January 2012). Total yeast count (left axis) is indicated as a black thick line (top of the graph). Temperature (right axis) is indicated as a dashed black line. The dashed arrows indicate when the yeast cell count rises to or drops below the detection limit [1.5 10^4^ colony forming units (CFU) g^−1^]. Only the four “core” yeast species are shown independently. The relative contribution of a yeast species (indicated by the colors) to the population at a certain time point is represented as the fraction (%) of the total yeast count. Note that the scale for the total yeast count (left) is logarithmic, whereas the relative fraction of each yeast species in the population is presented as fraction of the total population and thus represented on a linear scale. Solid arrows indicate turning. The second turning (96 h) of B2 and H2 is not shown.

Despite the large number of different yeasts found in the four fermentations, and despite that several species were only found in one or a few fermentations, a general trend emerges for the most prominent species. More specifically, four yeast species (*S. cerevisiae*, *H. thailandica*, *H. opuntiae* and *P. kudriavzevii*) form the “core yeast microbiota” that accounts for the vast majority of the yeast population in all fermentations (Figure 3). Of these, *S. cerevisiae* was the most dominant species in all fermentations. It was first detected after 14 h (H1, B2 and H2) or 22 h (B1), after which this species quickly became dominant, reaching maximum relative population sizes (close to) 100%. The yeast population that initially colonized the cocoa pulp consisted mainly of apiculate yeasts, most notably *H. thailandica*, representing 55% (H2) to 86% (H1) of the yeast population. However, its relative population size decreased after about one day. Similarly, *H. opuntiae* was mostly detected at the start of the fermentations and to a lesser extend in the middle of some fermentations, but this species was not dominating in any of the fermentations. *P. kudriavzevii* was only detected sporadically in B1 and H2, but constituted a considerable part of the yeast population in H1 and B2.

## Discussion

By following four independent fermentations that are carried out and analyzed in a standardized way by the same set of researchers, and by performing a detailed characterization of both bacterial and yeast species, we are able to directly compare the variation and consistency between the microbiota in different Malaysian cocoa pulp fermentations. In this way, we were able to establish a detailed overview of the microbial population dynamics in these spontaneous cocoa pulp fermentations in Malaysia, thereby covering the last important cocoa-producing region in the world.

Despite the use of two different fermentation systems (heap and box), and despite sampling on different time points in the main harvest season (early and late), the analyzed cocoa pulp fermentations show a remarkable consistency. All fermentations are dominated by the same bacterial and yeast species and show a similar global succession of these populations. Specifically, the core microbiota consists of the two dominant bacterial species, *L. fermentum* and *A. pasteurianus*, and four different yeast species, *S. cerevisiae*, *H. thailandica*, *H. opuntiae* and *P. kudriavzevii*. This core microbiota is probably favored by the highly selective environmental conditions present in cocoa pulp fermentations, especially at later stages of the process, such as high temperatures and the presence of chemical stressors, like ethanol, acetic acid and lactic acid. However, each of the fermentations also carries a variable microbiota. Interestingly, the variability of yeast species is much larger than that of bacteria. Although the population densities of these secondary yeast species are relatively limited, their presence or absence may have profound effects on the fermentation efficiency and end product quality, either directly or indirectly (e.g. through their influence on the core microbiota).

In all fermentations, *L. fermentum* is the dominating LAB species. The high acid, ethanol and heat tolerance of *L. fermentum* and the ability to convert citric acid (a major constituent of cocoa pulp and determinant of pH), provides the capacity to successfully dominate cocoa pulp fermentations in all cocoa-producing countries [13], [14], [15], [16], [17], [18]. Other species, such as *L. plantarum*, *Lc. pseudomesenteroides* and *L. brevis* are only observed in smaller quantities and often not in all fermentations. The AAB diversity is even more limited, with *A. pasteurianus* as the only species isolated from all fermentations. Therefore, *A. pasteurianus* and *L. fermentum* are considered as the bacterial core microbiota.

Although the different spontaneous fermentations are characterized by a large yeast diversity, the four core yeast species, *S. cerevisiae*, *H. thailandica*, *H. opuntiae* and *P. kudriavzevii*, consistently dominate the yeast population. The fast glucose-fermenting, Crabtree-negative *H. thailandica* dominates at the onset, and the ethanol and temperature tolerant *S. cerevisiae* becomes dominant at later time points. Nevertheless, a total number of 23 different species is detected, with the largest diversity being observed at initial stages of the fermentation. Most of these species were previously encountered in spontaneous cocoa pulp fermentations worldwide, but five of these species have, to our knowledge, never been associated with spontaneous cocoa pulp fermentations, namely *Candida asiatica*, *Candida fructus*, *C. metapsilosis*, *R. mucilaginosa* and *T. globosa*.

This study shows interesting similarities and differences with previous work. First, our analyses of the bacterial species are in line with previous reports, confirming the dominance of *L. fermentum* and *A. pasteurianus* [13], [14], [15], [16], [17], [18]. By contrast, the dominating yeast species seem to differ between studies. For example, this is the first report of *H. thailandica* as being a dominating yeast species. Additionally, while previous studies often report that the initial phases of cocoa pulp fermentations are dominated by *Hanseniaspora guilliermondii* [11], [12], [14], [46], we do not detect *H. guilliermondii* in Malaysian fermentations, but only its closely related species *H. opuntiae*. However, based upon ITS sequences, it was recently suggested that isolates from *H. opuntiae* have previously been misidentified as *H. guilliermondii* [26]. At later stages during the fermentation, *Pichia kudriavzevii* (teleomorph of *Candida krusei*) is reported to be the most dominant yeast in many studies [11], [12], [17], [28], but in our analyses its role is more limited. Similarly, *P. membranifaciens*, one of the most frequently isolated yeast species in cocoa pulp fermentations [11], [12], [14] is not found in this study. However, we do isolate *P. manshurica*, a closely related species. This latter species was also occasionally reported in previous studies [17], [26]. Finally, the most dominant yeast species in our analyses, *S. cerevisiae*, has been reported in cocoa pulp fermentations worldwide, but most of the studies detect *S. cerevisiae* in relatively low numbers, for example only at initial stages of the fermentations [11], [12], [17]. Together, our results (supplemented with results from previous research papers), hint towards the existence of a characteristic microbial succession in spontaneous cocoa pulp fermentations, consisting of the early dominance of fast-fermenting, but stress-sensitive apiculate yeasts, quickly outcompeted by more robust and stress-resistant species such as *S. cerevisiae* or *P. kudriavzevii*.

Our study also provides an important step towards the development of a good starter culture for cocoa pulp fermentations. Because of the highly variable qualitative outcome of spontaneous cocoa pulp fermentations, the development of starter cultures has received increasing interest. When developing an optimal microbial starter culture, it is essential to know which species are present and dominant in spontaneous fermentations, in order to select (beneficial) yeast and/or bacterial starter strains that are able to outcompete these spontaneous inocula. Our study highlights that the yeast microbiota is more variable than the bacterial microbiota, suggesting that inoculating a suitable yeast species might be sufficient to reduce the variability between different fermentations. In the attempt of simplifying starter cultures for cocoa pulp fermentations, a recent study investigated the use of a *L. fermentum-A. pasteurianus* starter culture [47]. Whereas using this culture accelerated the fermentation process regarding citric acid conversion and lactic acid production, the study concludes by stating that the addition of a yeast strain to the starter culture is an absolute requirement to overcome qualitative variability of the end product, since addition of a cocoa-specific *Saccharomyces* strain resulted in a more reproducible fermentation outcome. The combination of these results and the data presented in this paper, shows that *S. cerevisiae* is capable of dominating spontaneous cocoa pulp fermentations. Moreover, since *S. cerevisiae* is used as the preferred starter culture for most food and beverage fermentation processes and has therefore received the “generally recognized as safe” (GRAS) and “qualified presumption of safety” (QPS) status, this species seems an interesting candidate to be used in starter cultures for cocoa pulp fermentations. The use of *S. cerevisiae* as a starter culture has been described before [48]. Although conceptually interesting, commercial application of the tested *S. cerevisiae* strain was not achieved because of the lack of significant improvement in the fermentation process. This indicates that, due to the huge intraspecific diversity within *S. cerevisiae*, a well-considered screening and selection procedure is indispensable. Therefore, detailed characterizing and phenotyping indigenous *S. cerevisiae* strains, such as the big collection isolated in this study, will help to unravel the required characteristics for yeast strains to be used in efficient starter cultures and hence help us in selecting or developing an optimal yeast strain for controlled cocoa pulp fermentations.

## Supporting Information

Figure S1 **Dendrograms of (GTG)_5_-PCR fingerprints from 196 bacterial isolates that were identified as lactic acid bacteria.** A: box and heap fermentation from the start of the harvest season (October 2011). B: box and heap fermentation from the end of the harvest season (January 2012). Banding patterns were clustered using the unweighted pair group method with arithmetic mean algorithm and the Dice coefficient. A total number of 88 representative lactic acid bacterial strains were sequenced, indicated on the dendrograms. *L. brevis* = *Lactobacillus brevis*, *L. fermentum* = *Lactobacillus fermentum*, *L. plantarum* = *Lactobacillus plantarum*, *Lc. mesenteroides* = *Leuconostoc mesenteroides*, *Lc. pseudomesenteroides* = *Leuconostoc pseudomesenteroides*.
(TIF)

Figure S2 **Dendrograms of (GTG)_5_-PCR fingerprints from 170 bacterial isolates that were identified as acetic acid bacteria.** A: box and heap fermentation from the start of the harvest season (October 2011). B: box and heap fermentation from the end of the harvest season (January 2012). Banding patterns were clustered using the unweighted pair group method with arithmetic mean algorithm and the Dice coefficient. A total number of 30 representative acetic acid bacterial strains were sequenced, indicated on the dendrograms. *A. pasteurianus* = *Acetobacter pasteurianus*.
(TIF)
