# Supplementary figures and images for: Detailed Analysis of the Microbial Population in Malaysian Spontaneous Cocoa Pulp Fermentations Reveals a Core and Variable Microbiota
Source: PLoS One. 2013 Dec 16;8(12):e81559. doi: 10.1371/journal.pone.0081559 (PMC3864809; doi:10.1371/journal.pone.0081559)

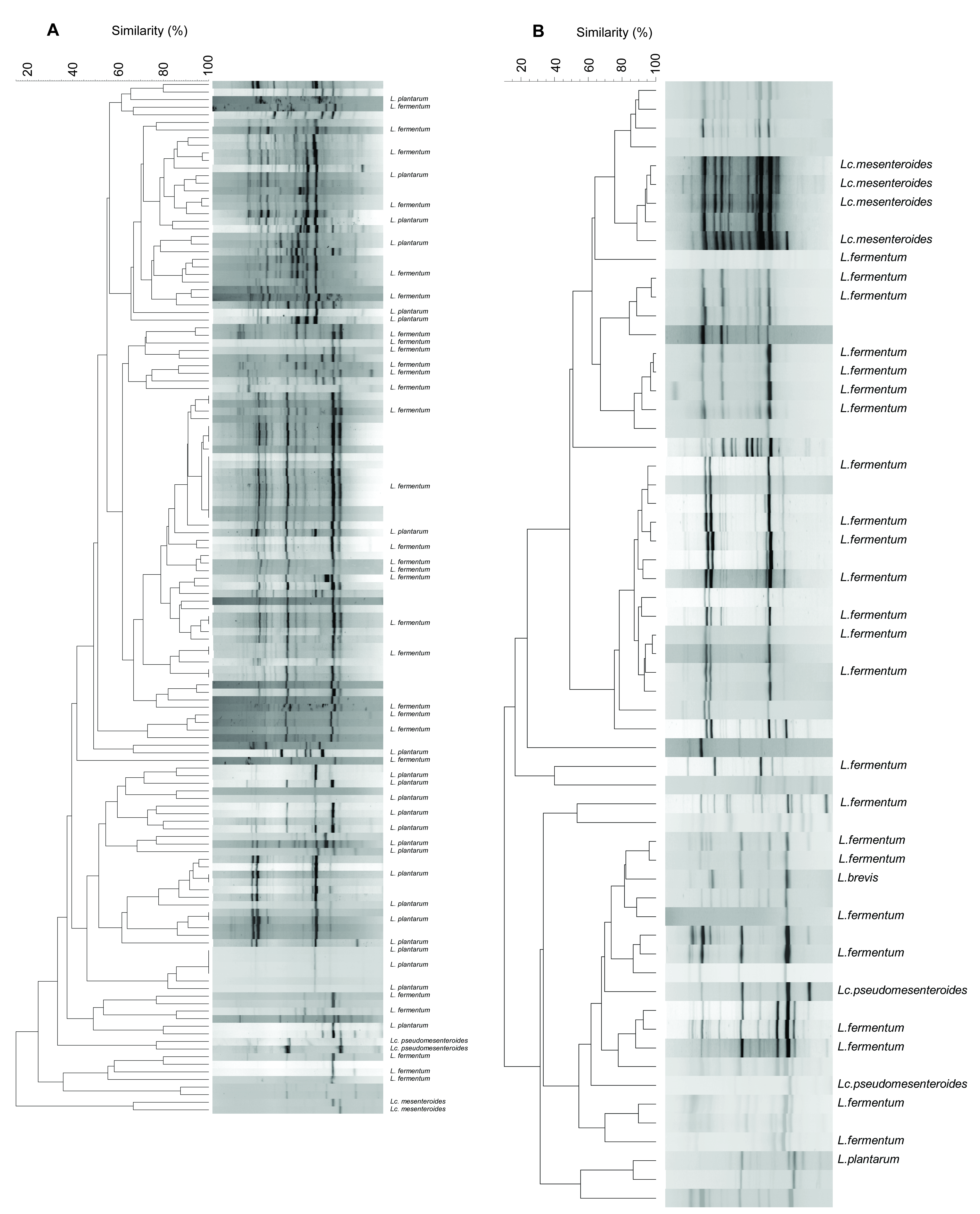

Supplement: Figure S1 — Dendrograms of (GTG)5-PCR fingerprints from 196 bacterial isolates that were identified as lactic acid bacteria. A: box and heap fermentation from the start of the harvest season (October 2011). B: box and heap fermentation from the end of the harvest season (January 2012). Banding patterns were clustered using the unweighted pair group method with arithmetic mean algorithm and the Dice coefficient. A total number of 88 representative lactic acid bacterial strains were sequenced, indicated on the dendrograms. L. brevis = Lactobacillus brevis, L. fermentum = Lactobacillus fermentum, L. plantarum = Lactobacillus plantarum, Lc. mesenteroides = Leuconostoc mesenteroides, Lc. pseudomesenteroides = Leuconostoc pseudomesenteroides. (TIF) [file pone.0081559.s001.tif]

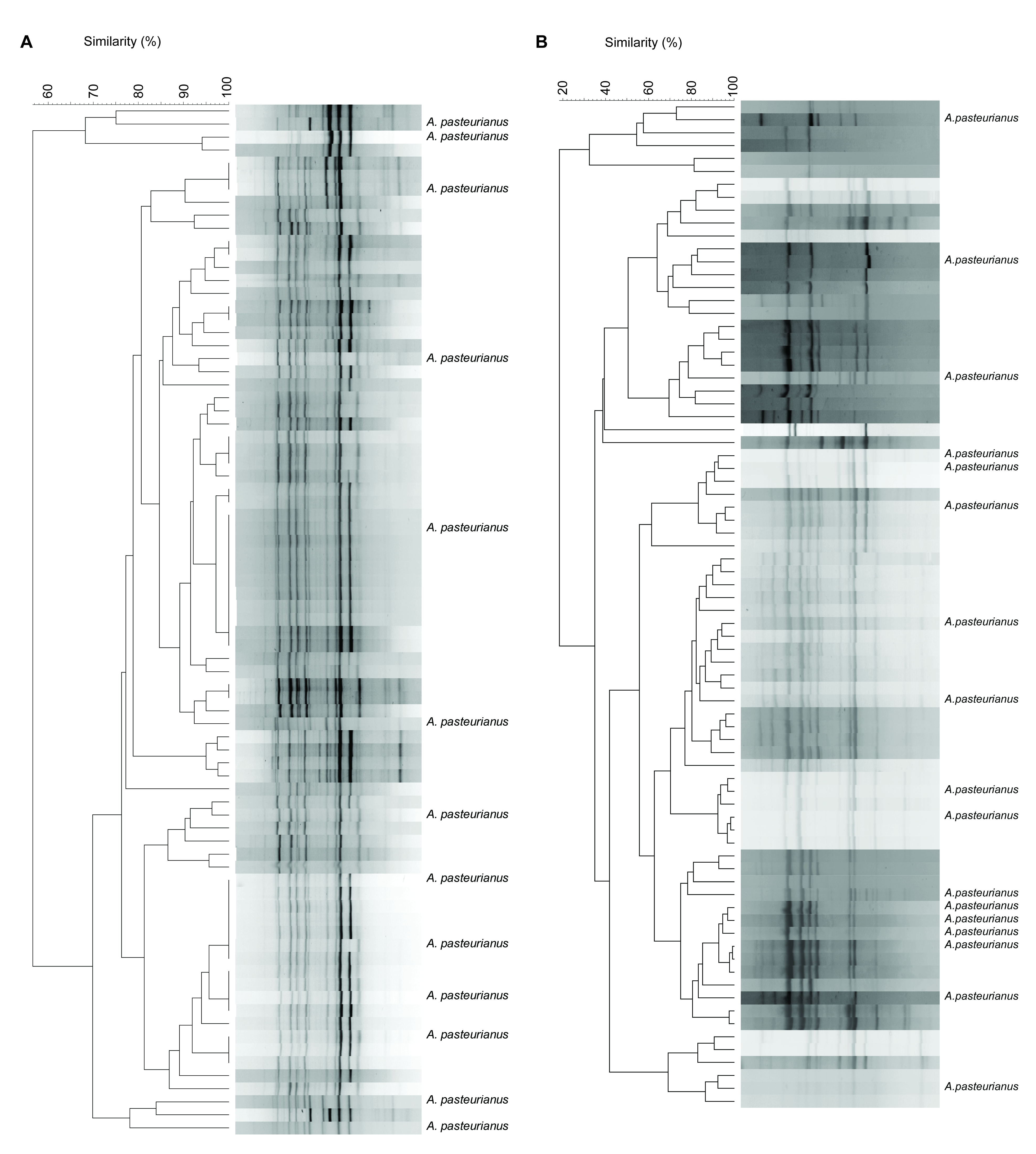

Supplement: Figure S2 — Dendrograms of (GTG)5-PCR fingerprints from 170 bacterial isolates that were identified as acetic acid bacteria. A: box and heap fermentation from the start of the harvest season (October 2011). B: box and heap fermentation from the end of the harvest season (January 2012). Banding patterns were clustered using the unweighted pair group method with arithmetic mean algorithm and the Dice coefficient. A total number of 30 representative acetic acid bacterial strains were sequenced, indicated on the dendrograms. A. pasteurianus = Acetobacter pasteurianus. (TIF) [file pone.0081559.s002.tif]
